# Multiplex Detection and Quantification of miRNAs in Drug Delivery Systems Using a Signal-Off Electrochemical Platform

**DOI:** 10.1021/acs.analchem.5c07348

**Published:** 2026-04-27

**Authors:** Wanda Cimmino, Alessia Angelillo, Panagiota M. Kalligosfyri, Valeria Nele, Virginia Campani, Stefania Carbone, Concetta Di Natale, Giuseppe De Rosa, Stefano Cinti

**Affiliations:** † Department of Pharmacy, 9307 University of Naples Federico II, Naples 80131, Italy; ‡ Department of Life Science, Health and Health Professions, 207131 Link Campus University, Rome 00165, Italy; § Department of Chemical, Materials and Industrial Production Engineering, University of Naples Federico II, Piazzale V. Tecchio 80, Naples 80131, Italy; ∥ Bioelectronics Task Force at University of Naples Federico II, Via Cinthia 21, Naples 80126, Italy; ⊥ Sbarro Institute for Cancer Research and Molecular Medicine, Center for Biotechnology, College of Science and Technology, Temple University, Philadelphia, Pennsylvania 19122, United States; # Department of Chemistry, Faculty of Science, Chulalongkorn University, Bangkok 10330, Thailand

## Abstract

Lipid nanoparticles (LNPs) are widely used for the delivery of therapeutic microRNAs (miRNAs), especially in coformulated systems where multiple targets are combined to enhance therapeutic efficacy. However, the simultaneous quantification of miRNAs within LNP formulations remains a major analytical challenge for pharmaceutical quality control. This work presents a dual-working electrochemical biosensor for multiplexed, selective, and amplification-free quantification of two miRNAs (miR-4676 and miR-6503) encapsulated in LNPs. The biosensor was fabricated on a dual-working screen-printed graphite electrode via gold electrodeposition, followed by site-specific immobilization of methylene blue (MB)-labeled ss-DNA probes. After surfactant-mediated LNP lysis, square wave voltammetry (SWV) enabled hybridization-based signal-off detection of the released targets. The sensor showed excellent analytical performance, with limits of detection of 0.96 nM for miR-4676 and 0.98 nM for miR-6503. High selectivity was confirmed through cross-reactivity experiments. In real formulations with different miRNA ratios (1:1 and 2:1), the sensor achieved quantification accuracies of 88% and 84%, respectively, compared to a standard fluorimetric assay. Unlike conventional methods, the platform enabled individual miRNA quantification in complex mixtures. This study introduces an electrochemical platform enabling multiplexed miRNA quantification within LNPs, providing a rapid, low-cost, and portable solution for quality control of miRNA-based formulations.

## Graphical abstract

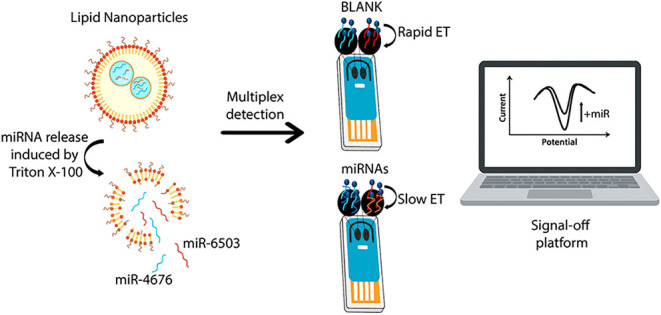

## Introduction

The rapid advancement of RNA therapeutics has established lipid nanoparticles (LNPs) as a key platform for efficient nucleic acid delivery. Owing to their structural versatility, biocompatibility, and proven clinical performance, as demonstrated by mRNA vaccines, LNPs are widely used for the encapsulation and systemic administration of RNA molecules.^1^ Among these, miRNAs represent a promising class of therapeutic agents, capable of modulating gene expression with high specificity, and are being explored in numerous disease areas including cancer, cardiovascular diseases, and neurodegenerative disorders.^2^

Recent advances in the codelivery of multiple miRNAs within a single LNPs formulation have demonstrated enhanced therapeutic efficacy by targeting synergistic molecular pathways.^3^ In this context, LNPs are increasingly explored as versatile carriers for multicargo strategies, including the coencapsulation of two or more miRNAs, motivating the need for analytical tools capable of individually quantifying each RNA component within a single formulation. These multi-miRNA formulations are increasingly applied in oncology and inflammatory diseases, where simultaneous modulation of multiple genes offers improved clinical outcomes.^4^ However, their development introduces new challenges in the quality control process, as each component must be accurately quantified to ensure proper dosing and batch-to-batch consistency.^5^

These earlier efforts addressed critical issues in single-miRNA quantification and laid the foundation for the development of more advanced systems capable of handling multiple targets.

Current analytical methods for LNPs characterization, such as reverse transcription quantitative PCR (RT-qPCR), fluorescence assays and HPLC are highly sensitive and well-established. However, they are often limited by high operational complexity, cost, and incompatibility with high-throughput or in-line quality control workflows.^6^ These challenges are particularly evident when analyzing complex, coencapsulated miRNA formulations, which require multiplexed detection and quantification of multiple RNA targets within a single assay.^7^ Therefore, there is a growing need for the development of multiplexed, cost-effective, and field-deployable analytical tools that can meet the demands of nowadays LNPs characterization, particularly in resource-limited or fast-paced production environments.

Electrochemical biosensors have emerged as a promising alternative for nucleic acid detection due to their simplicity, affordability, and adaptability to miniaturized formats.^8,9^ Recent studies have shown their capability to detect miRNAs at femtomolar concentrations, often leveraging optimized probe immobilization strategies and nanostructured interfaces.^10^ Nonetheless, most electrochemical approaches are limited to single-analyte detection. While some multiplexed systems have been proposed, employing dual redox labels,^11^ hybridization chain reaction (HCR),^12^ or digital PCR platforms,^13^ they often require complex instrumentation or are not compatible with lipid-based matrices.

Our group has contributed to this field by employing electrochemical sensing strategies to the quantification of model analytes such as methylene blue (MB) in lipid nanovesicles (LNVs),^14^ and for the detection of miRNAs encapsulated in LNPs.^15^

Building on these previous efforts, our earlier work demonstrated that electrochemical biosensing can be successfully applied to quantify a single miRNA encapsulated in LNPs.^15^ However, as RNA therapeutics continue to evolve, modern formulations increasingly rely on the codelivery of multiple miRNAs within a single LNPs system to enhance therapeutic outcome through synergistic regulation of biological pathways. This trend is becoming particularly relevant in cancer therapy and inflammatory diseases, where dual-miRNA administration has shown superior efficacy and reduced resistance mechanisms.^16^

These advancements introduce a new analytical challenge: single-analyte assays are no longer sufficient for robust pharmaceutical quality control, since each RNA component must be individually quantified to ensure correct dosing and batch consistency. To date, electrochemical approaches enabling multiplexed and sequence-resolved quantification of multiple miRNA species within the same LNP formulation, specifically tailored to pharmaceutical quality control applications, have not been reported in the literature. This lack of multiplexing capability limits the ability to translate biosensing technologies to real-world production and release-testing pipelines.

In this work, we address this unmet analytical need by developing a multiplexed electrochemical biosensing platform specifically designed for the quality control of dual-miRNA LNPs formulations. The novelty of the proposed approach does not lie in the signal-off transduction mechanism itself, which is well established for nucleic acid sensing, but rather in its application to sequence-resolved, dual-target quantification of coencapsulated miRNAs within pharmaceutical LNPs formulations.

Unlike conventional fluorimetric assays such as RiboGreen and standard RT-qPCR approaches, which provide total RNA quantification or require complex workflows for sequence-specific analysis, the proposed platform enables the independent quantification of each miRNA species within the same formulation. This capability is essential for ensuring correct dosing, formulation reproducibility, and batch-to-batch consistency in multi-miRNA therapeutics.

To the best of our knowledge, this is the first electrochemical biosensing strategy specifically developed for multiplex, sequence-specific quantification of multiple miRNA cargos within a single LNPs formulation for pharmaceutical quality control purposes.

Nowadays dual-drug/miRNA and dual-miRNA delivery systems have been explored.^17–20^ This motivated the selection of miR-4676 and miR-6503 for simultaneous detection, reflecting emerging therapeutic strategies. The device integrates two independently functionalized gold-modified working electrodes within a single screen-printed system, selected to combine the robustness of gold–thiol probe immobilization with the low cost, disposability, and scalability required for practical quality control applications. Each working electrode is equipped with a MB-labeled thiolated DNA probe selective for either miR-4676 or miR-6503. These two target were selected as representative model miRNA cargos to demonstrate sequence-resolved multiplexed quantification within coformulated LNPs systems. Signal discrimination in the proposed platform is achieved through spatial separation, as each miRNA target is detected on an independently functionalized working electrode. As illustrated in Figure 1, following LNPs lysis, the released miRNAs hybridize with their complementary probes, generating a signal-off voltammetric response that selectively reflects the concentration of each target.

**Figure 1:**
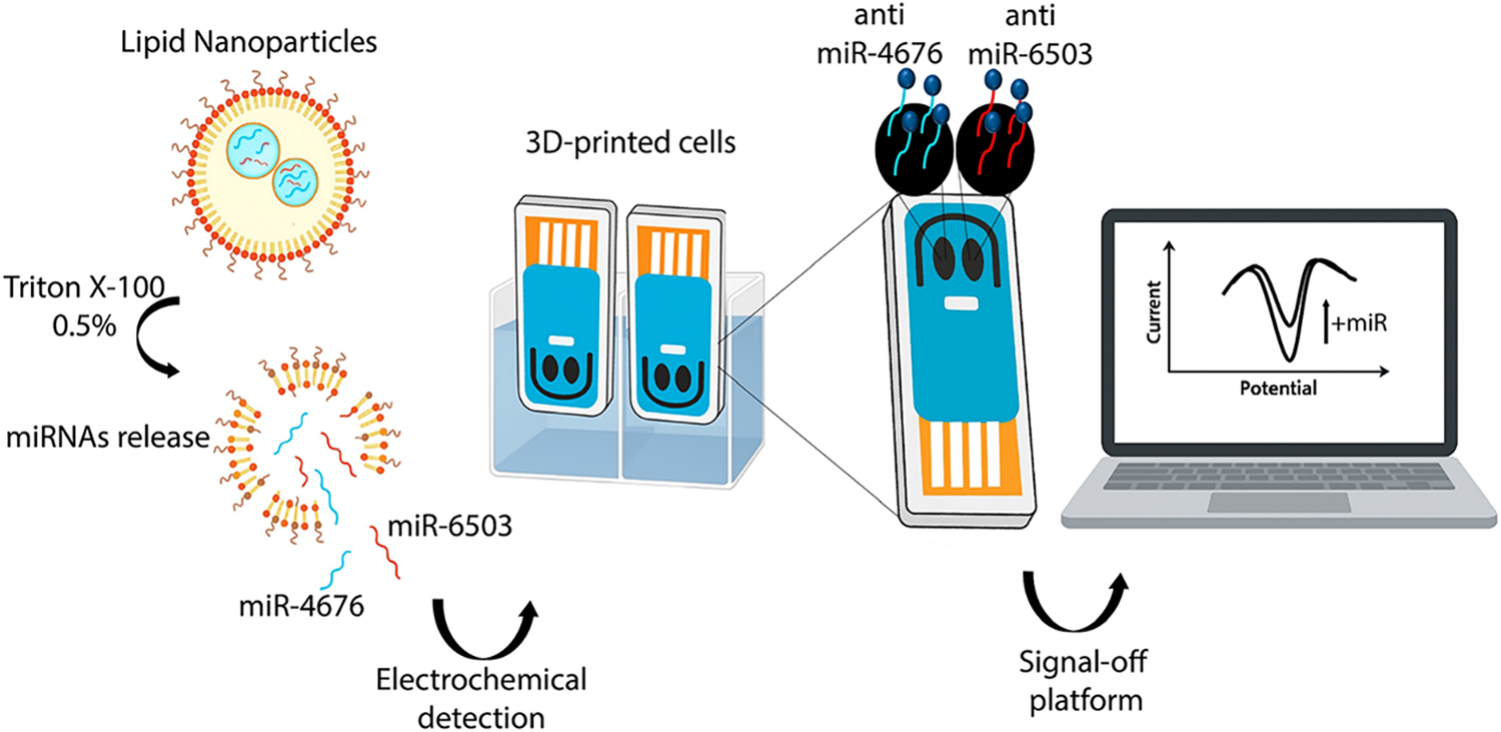
Schematic representation of the dual electrochemical biosensing strategy for miRNA quantification in LNPs formulations.

This architecture allows parallel, sequence-specific detection in the same measurement, eliminating the need for enzymatic amplification or multistep workflows. Furthermore, the platform maintains excellent analytical performance in both lipid-based matrices and human serum, demonstrating readiness for practical implementation in resource-limited or fast-paced production settings.

To our knowledge, this work represents the first application of a multiplexed electrochemical biosensing strategy to the quantitative analysis of coencapsulated miRNAs within a single LNPs formulation, specifically addressing pharmaceutical quality control requirements. Owing to its miniaturization, low cost, compatibility with standard handling procedures, and potential for automation, the proposed platform constitutes a substantial advancement toward scalable, real-time, and target-resolved quality control of emerging multi-miRNA RNA therapeutics.

## Experimental Section

### Chemicals and Materials

All the information regarding the chemical reagents and materials utilized are reported in the Supporting Information (SI).

### Biosensor Fabrication on Screen-Printed Dual Working Electrodes

Graphite screen-printed dual working electrodes were selected as the base sensing platform to enable low-cost, disposable, and scalable device fabrication. To ensure robust thiol-based probe immobilization and signal stability under surfactant-containing conditions required for LNPs disruption, the graphite working electrodes were modified by gold electrodeposition. The first step in the biosensor fabrication involved gold electrodeposition on the working electrodes (WEs) by chronoamperometry in a 25 mM HAuCl_4_ solution. The deposition parameters were: equilibration time (*T*_eq_) of 5 s, deposition potential (*E*_dc_) of −0.5 V, interval time (*T*_int_) of 0.1 s, and total deposition time (*T*_run_) of 300 s. The gold-modified working electrodes were used directly for probe immobilization. No additional electrochemical cleaning or activation in acidic media (e.g., H_2_SO_4_ cycling) was performed after electrodeposition. This choice is justified by the fact that the sensing interface is based on freshly electrodeposited nanostructured gold onto a graphite screen-printed substrate rather than bulk gold electrodes. The adopted protocol, previously validated in our recent work,^21^ provides reproducible and electrochemically active surfaces suitable for biosensing applications without the need for further electrochemical activation. Following this step, the working electrodes were modified according to a protocol optimized in our previous studies.^15,22,23^

Initially, the MB-ssDNA probes underwent disulfide bond (S–S) reduction using 0.01 M TCEP in the dark for 1 h. After reduction, each probe (anti-miR-6503 and anti-miR-4676) was diluted in PBS from the concentration of 1000 nM to the concentration of 100 nM.^24^ Then, a 5 μL drop of each probe was separately applied onto one of the two distinct working electrodes, ensuring that the two drops did not mix. This was followed by 1-h incubation in a humidity chamber to promote probe immobilization. After incubation, the electrodes were gently rinsed with deionized (DI) water. Subsequently, both working electrodes were incubated with a 2 mM MCH solution for 1.5 h in the humidity chamber to block nonspecific binding sites, followed by a final rinse with DI water.

The biosensor functions as a signal-off platform: in the presence of the miRNA target, hybridization with the immobilized MB-DNA probe reduces the electrochemical accessibility of the MB, resulting in a decrease in current signal. Thus, the current response decreases in the presence of the target compared to the blank.

All results are reported as percentage signal change, calculated by comparing the current in the absence of the target with the current obtained at different concentrations of the two miRNA targets.

### Surface Morphological and Elemental Characterization (SEM–EDX)

Scanning Electron Microscopy (SEM) images were acquired to investigate the surface morphology of the electrochemical sensors, both the bare electrode and the electrode modified with 2.5, 10 25 and 50 mM gold. The specimens were introduced into a Hitachi TM3000 tabletop SEM (Hitachi High-Tech, Tokyo, Japan) without prior metallization, to preserve the true surface state. Imaging was performed under standard operating conditions at two magnifications corresponding to scale bars of 200 and 100 μm. In addition, Energy Dispersive X-ray spectroscopy (EDX) was employed to monitor the elemental composition of the surfaces. Point-analysis spectrum was recorded at representative regions of each electrode type. The weight percentages of relevant elements (e.g., Au, Cl, O, Br, Si, Ti, Mg) were derived from the manufacturer’s quantification software.

### Electrochemical Measurements

Electrochemical measurements were carried out at room temperature using the dual SPEs placed in custom 3D-printed electrochemical chambers. Both the incubation and detection steps were performed directly within these chambers, ensuring consistent measurement conditions throughout the assay. All electrochemical measurements were performed after a lysis step and not on intact LNPs. Triton X-100 was employed to induce complete disruption of the LNPs and release of the encapsulated miRNAs prior to detection. Following lysis, samples were diluted 1:500, resulting in a final Triton X-100 concentration of 0.001% (v/v) in the electrochemical cell, a condition that does not affect the hybridization efficiency or the signal-off baseline. The complete dissolution of the LNPs and full release of the encapsulated miRNAs were ensured under these conditions. This treatment enables total nanoparticle disruption without affecting the hybridization efficiency of the DNA probes.^15^ Triton X-100 was employed as surfactant to induce complete disruption of LNPs and release of the encapsulated miRNAs prior to electrochemical analysis. The effectiveness of Triton-mediated LNPs lysis for total nucleic acid release is well established in the literature and is routinely adopted in standard RNA quantification protocols, including fluorimetric assays such as RiboGreen. In addition, in our previous work,^14^ the efficiency of LNPs disruption by Triton X-100 was systematically evaluated using a Design of Experiments approach, including temperature as factor, and no statistically significant improvement in miRNA release was observed upon thermal treatment in the presence of Triton. To further confirm release efficiency under the selected conditions, miRNA content following Triton treatment was independently assessed using a RiboGreen assay, yielding values consistent with the nominal formulation concentrations. The electrochemical detection of the released target was performed using square wave voltammetry (SWV) with the following parameters: equilibration time = 5 s, initial potential = +0.05 V, final potential = −0.6 V, step potential = 0.001 V, amplitude = 0.03 V, and frequency = 20 Hz.

The analytical signal was defined as the change in the peak current (Δ*I*), calculated as the difference between the SWV current recorded before and after hybridization with the target miRNAs. This signal decrease is attributed to the formation of the DNA–miRNA duplex at the electrode surface, which hinders electron transfer of the redox probe. The dual-working-electrode configuration enables multiplexed detection of two miRNA targets from the same lysed and diluted sample aliquot under identical experimental conditions. Each working electrode was independently functionalized with a specific probe and the electrochemical signals were recorded sequentially from two physically separated electrodes within the same device. A single sample volume of 100 μL is sufficient to interrogate both working electrodes within the same electrochemical chamber. In contrast, performing the same analysis using two independent single-target electrodes would require twice the sample volume and additional handling steps. This architecture therefore reduces sample consumption, experimental variability, and handling-related errors, which is particularly advantageous in pharmaceutical quality-control workflows.

### Preparation of LNPs Co-Encapsulating miR-4676 and miR-6503

LNPs coencapsulating miRNA miR-4676 and miR-6503 were prepared using the ethanol injection method.^25^ Briefly, an ethanol stock solution containing SM-102/CHOL/DSPC/DMG-PEG2000 (50:38.5:10:1.5 mol %) was prepared alongside a 10 mM citric acid solution (pH 4.0) containing both miRNAs. For coencapsulation, miR-4676 and miR-6503 were combined in either a 1:1 or 2:1 molar ratio, resulting in distinct LNP formulations. The lipid ethanol solution was then added dropwise to the aqueous miRNA solution under stirring in a 1:1.5 v/v ratio (N/P = 6). The resulting preparations were dialyzed (20 kDa cutoff) against 10 mM citrate buffer (pH 4.0 for 1 h) to remove the excess ethanol, and then against PBS 1× pH 7.4 overnight to remove the citrate buffer and neutralize the LNP surface charge. LNPs were concentrated using Amicon filters with a molecular weight cutoff of 3.5 kDa. Empty LNPs were also prepared and used as control samples.

### LNPs Physicochemical Characterization

The formulations were characterized in terms of colloidal dimensions, polydispersity index (PDI), and surface charge by using dynamic light scattering (DLS) (Zetasizer Nano Z, Malvern, U.K.). For each formulation the z-average diameter, PDI, and zeta potential were calculated as mean value ± standard deviation of the measurements from *N* = 3 independent batches.

### Lipid Dosage in LNPs

The amount of phospholipids in the LNP miR-6503 and miR-4676 was determined by the Stewart assay.^25^ Briefly, an aliquot of the LNPs was added to a two-phase system, consisting of an aqueous ammonium ferro thiocyanate solution (0.1 N) and chloroform. Each tube was mixed on vortex and then centrifugated (Hettich UNIVERSAL 320 R, Andreas Hettich GmbH) at 1000 rpm for 10 min. The chloroform phase was collected and the concentration of DSPC was obtained by measure of the absorbance at 485 nm with an ultraviolet–visible spectrophotometer (UV VIS 1204; Shimadzu Corporation, Kyoto, Japan). The concentration of the total lipids content was calculated considering a constant ratio between the lipids.

### Assessment of Encapsulation Efficiency of the miR-4676 and miR-6503 in LNPs

The miRNA encapsulation efficiency was quantified by fluorimetric assay using the RiboGreen RNA Quant-iT Assay kit (Thermofisher); for this purpose, the formulations are diluted in Tris-EDTA (TE) 1× buffer containing 1% v/v Triton-X (permeabilized LNPs) or TE 1× buffer (intact LNPs). A miRNA standard curve (*R* ^2^ = 0.99) was used. The miRNA encapsulation efficiency was determined as follows

$$e\mathrm{ncapsulation efficiency}\left(\%\right)=\frac{{\left[\mathrm{miRNA}\right]}_{\mathrm{total}}-{\left[\mathrm{miRNA}\right]}_{\mathrm{unencapsulated}}}{{\left[\mathrm{miRNA}\right]}_{\mathrm{total}}}\times 100$$

where [miRNA]_total_ is the total miRNA concentration in the formulation and [miRNA]_unencapsulated_ is the concentration of unencapsulated miRNA. Since the assay measures the total rather than individual miRNA species, calculations are based on the assumption that the initial molar ratio between miR-4676 and miR-6503 is maintained throughout the formulation process.

## Results and Discussion

### Physicochemical Characterization of Lipid Nanoparticles

All LNPs are characterized as described in the Materials and Methods section. Both empty LNPs and those coencapsulating miR-4676 and miR-6503 (at 1:1 or 1:2 molar ratios) display a hydrodynamic diameter below 200 nm, a low polydispersity index (PDI < 0.2), and an approximately neutral pH. In both formulations, the encapsulation efficiency is close to 80%, calculated as explained in the section “assessment of encapsulation efficiency of miR-4676 and miR-6503”, while the total lipid concentration is 3.5 mg/mL. The corresponding graphs are available in the SI (Figure S1).

### Surface Morphology and Elemental Characterization of Gold-Deposited Electrodes (SEM–EDX)

Full SEM and EDX characterization confirming the uniformity and efficiency of the gold deposition is provided in the SI (Figure S2).

### Optimization of Experimental Parameters

#### Gold Electrodeposition Optimization

In a previous work, the influence of Triton X-100 on the hybridization and on the stability of ss-DNA on the electrode surface was investigated using gold-based electrodes.^15^ In the present study, however, graphite electrodes were selected as platform. To enable probe immobilization and ensure signal retention in surfactant-rich environments, a gold electrodeposition step was introduced and optimized. Gold electrodeposition optimization was performed in the presence of 0.5% Triton X-100, corresponding to LNPs lysis, and not to the final diluted Triton concentration used during miRNA quantification. This approach was adopted as a stress-test to identify gold deposition conditions capable of ensuring probe stability and signal retention even in the presence of high detergent concentrations.

Unlike drop-cast gold nanoparticles, electrochemically deposited gold offers greater surface homogeneity and adhesion.^26,27^ Consistently, the electrodeposited surface exhibits a higher areal capacitance (147.60 μF cm^–2^) than both the drop-cast AuNPs-modified electrode (130.18 μF cm^–2^) and the bare electrode (112.36 μF cm^–2^), suggesting a larger electroactive surface area due to improved surface coverage and reduced nanoparticle aggregation. The aim was to identify the optimal deposition condition that would preserve the performance of the signal-off biosensor even in the presence of Triton X-100. For this purpose, four different concentrations of HAuCl_4_ (2.5, 10, 25, and 50 mM) were tested.

After the immobilization of 100 nM of probe, on the electrode surface, we performed the experiments, in the presence of 20 nM of miRNA target, both in the absence of Triton X-100 and under lysis conditions (0.5% Triton X-100) (Figure 2A).

**Figure 2:**
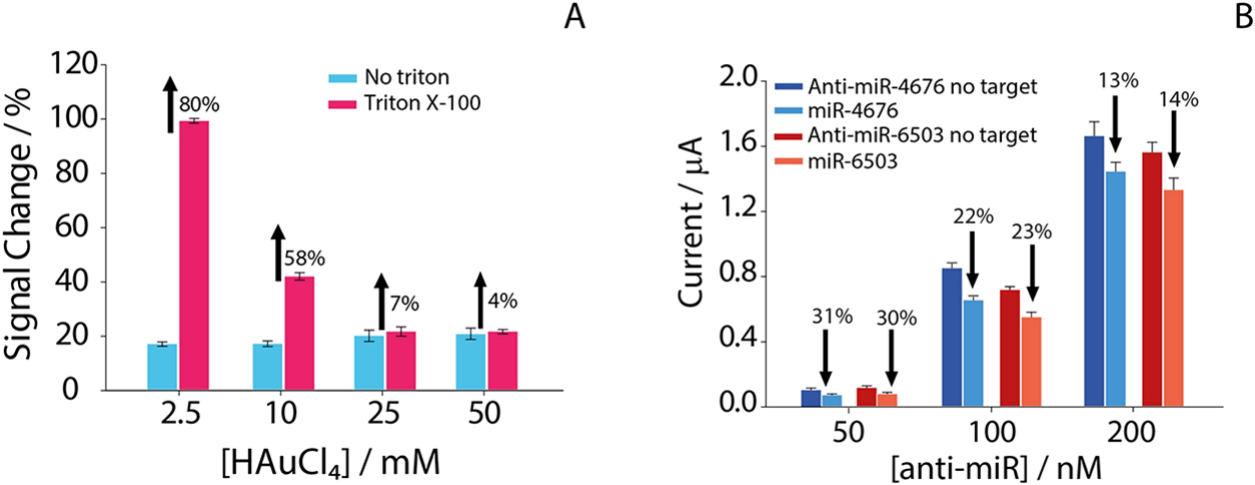
(A) Effect of Triton X-100 on the signal change (%) at different concentrations of electrodeposited HAuCl_4_ (2.5, 10, 25, and 50 mM). Blue bars signal change % obtained in the presence of 20 nM of miRNA target in the absence of Triton X-100, pink bars signal change % obtained in the presence of 20 nM of miRNA target and Triton X-100. The percentage differences between the two conditions are indicated above each pair of bars. All the experiments were conducted in the presence of 100 nM of probe, 0.5% of Triton X-100 and in triplicates. (B) Effect of anti-miR probe concentration (50, 100, and 200 nM) on the electrochemical response of biosensor in the presence of 50 nM target miRNA. Dark-colored bars represent the current recorded after probe immobilization (blank), while light-colored bars correspond to the current measured after hybridization with the miRNA target. The signal change % induced by hybridization is reported above each pair of bars.

In signal-off biosensing systems, hybridization with the target typically leads to a reduction in the signal intensity. To establish a baseline for comparison, the signal recorded in the absence of Triton was considered the reference, representing the optimal hybridization condition. In this state, the hybridization process occurs without any interference from detergents or disruptors, ensuring that the measured signal accurately reflects the full binding efficiency of the target. The signal change observed in the presence of Triton X-100 was attributed to surface destabilization or interference, providing an indication of the effect of the detergent on the system’s integrity. As shown in the Figure 2, at low concentrations (2.5 and 10 mM), substantial signal suppression was observed in the presence of Triton X-100, with current losses of 80% and 58%, respectively. A significant improvement was observed at 25 mM HAuCl_4_, with only 7% signal variation compared to Triton free reference sample. Increasing the gold concentration to 50 mM resulted in a minimal additional gain (4%). These findings are consistent with the compositional analysis reported in Figure S3, which shows a progressive increase in Au weight percentage with increasing HAuCl_4_ concentration (from ∼28.3 wt % at 2.5 mM to ∼98.9 wt % at 50 mM). This trend indicates a gradual increase in gold surface coverage, supporting the hypothesis that the improved resistance to Triton X-100 observed at higher concentrations is associated with the formation of a more continuous and stable gold layer. At lower gold loadings (2.5 and 10 mM), the partial surface coverage likely results in less robust probe immobilization, making the system more susceptible to surfactant-induced destabilization. In contrast, at 25 mM, the high gold content suggests the formation of a sufficiently dense and homogeneous gold surface, which enhances probe anchoring and preserves the electrochemical response even under lysis conditions. The marginal improvement observed at 50 mM is consistent with a near-saturation of the surface, as also indicated by the EDX data.These results confirm that 25 mM gold electrodeposition provides sufficient surface coverage to preserve probe stability and analytical performances even in the presence of surfactants (Figure S2). This condition was therefore selected for all subsequent experiments. The electrochemical behavior of the electrodes before and after gold electrodeposition at the selected condition (25 mM HAuCl_4_) was further evaluated by cyclic voltammetry, as reported in the SI (Figure S4).

Following gold optimization, the concentration of the immobilized probe was investigated to maximize the analytical response while maintaining adequate signal intensity and repeatability. Probe concentrations of 50, 100, and 200 nM were evaluated using a fixed target concentration of 50 nM miRNA (Figure 2B).

As shown in Figure 2B, in the presence of 50 nM probe, the highest percentage change in signal was obtained after hybridization, indicating a high relative sensitivity of the platform. However, the absolute current values recorded at this probe concentration were too low (∼0.1 μA), resulting in increased signal variability and reduced repeatability. In contrast, increasing the probe concentration to 100 nM resulted in higher and more stable current responses, while maintaining a pronounced and reproducible signal decrease during hybridization. A further increase in probe concentration to 200 nM resulted in a further increase in current, accompanied by a reduction in relative signal variation, probably due to steric hindrance and limited target accessibility at high surface densities.^22^

Considering the trade-off between signal amplitude, percentage signal variation and repeatability, a probe concentration of 100 nM was selected as the optimal condition for subsequent analytical measurements. This choice ensures a robust and reproducible electrochemical response, which is essential for reliable quantification of miRNAs in complex matrices.

### Analytical Performances, Selectivity Study and miRNA Quantification

To validate the analytical performances of the dual- working electrode biosensor, a series of control and quantification experiments were conducted. All the samples were treated with 0.5% (v/v) Triton X-100 to induce the lysis of LNPs,^15,28,29^ this step was followed by a 1:500 dilution step to ensure that the miRNA concentration remained in the linear response range of the biosensor. After dilution, the final concentration of Triton X-100 in the electrochemical cell was 0.001% (v/v). This protocol was applied identically to all the samples. The first step was the evaluation of the biosensor performances, they were tested constructing a binding curve in real matrix, using empty LNPs, spiked with different concentrations (ranging from 0.1 to 800 nM) of the two miRNAs target. As shown in the Figure 3a characteristic sigmoidal correlation is shown between the miRNA concentration and the signal change %. The curves show a good correlation with a *R* ^2^ of 0.99. The data were fitted using a four-parameter Hill model, described by the equation: $Y=3.43+\frac{72.01\times x^{0.78}}{81.30^{0.78}+x^{0.78}}$ for miR-6503 and $Y=2.97+\frac{84.30\times x^{0.87}}{100.49^{0.87}+x^{.0.87}}$ for miR4676. The limit of detection (LOD) was calculated from the fitted sigmoidal curves by considering the lower asymptote (*y*_0_) as the blank response and the standard error associated with y_0_ as an estimate of the baseline noise (σ). The signal corresponding to the LOD was calculated as *y*LOD= *y*_0_ + 3σ, and the corresponding concentration vlue was obtained by bsck-calculating the inverse Hill equation. Using this approach, the LOD values were found to be 0.96 nM for miR-6503 and 0.98 nM for miR-4676. Although the LOD values are in the low nM range, they are fully adeguate for the intended application. In LNPs formulations, encapsulated miRNAs are typically present at μM concentrations.

**Figure 3:**
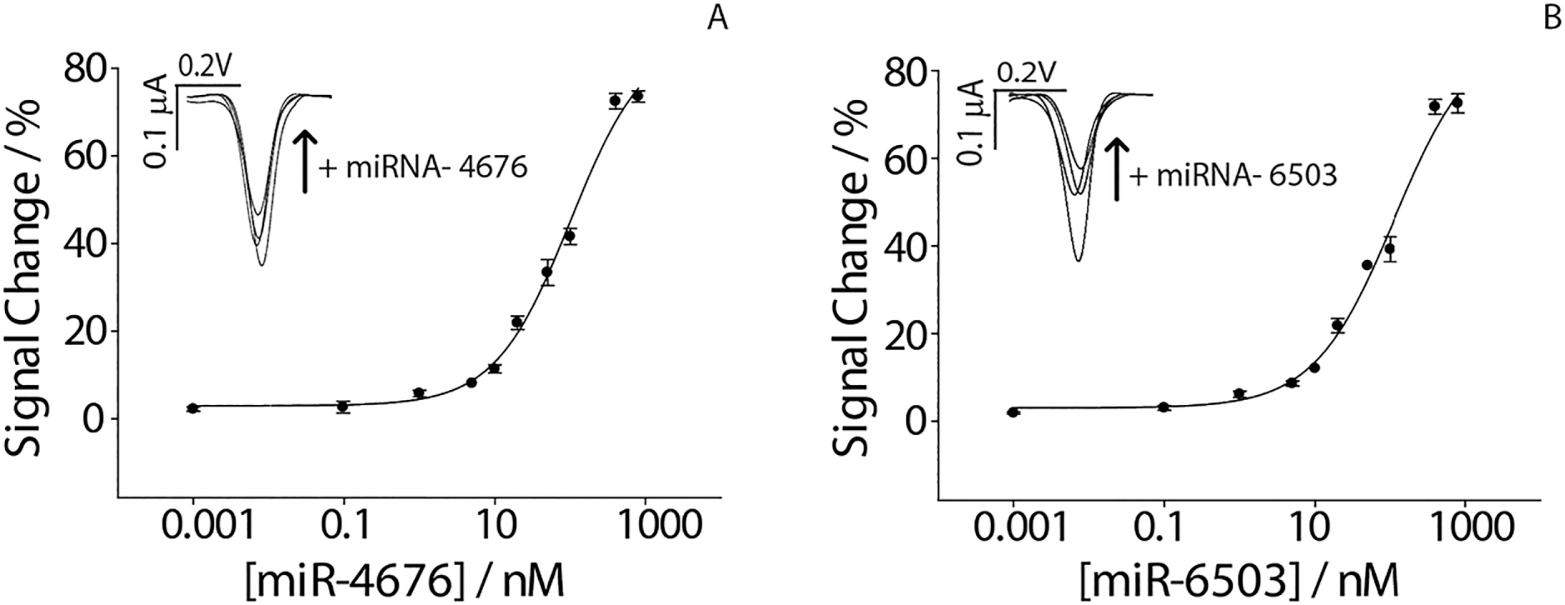
Binding curves obtained in empty LNPs spiked with different concentrations of miRNAs ranging from 0.1 nM to 800 nM. (A) Binding curve obtained testing miR-4676. (B) Binding curve obtained testing miR-6503. The insets of each figure show the signal off voltametric curves corresponding to the increase of miRNAs. All the measurements were performed in triplicate and in the presence of Triton X-100.

For a clearer positioning of the proposed platform with respect to established methods for miRNA detection, we compared its main analytical and practical features (LOD, assay time and costs) with those of RT-qPCR and the Quant-iT RiboGreen assay. This comparison, reported in Table S1 (SI), highlights the trade-off between sensitivity and operational simplicity that makes the present biosensor suitable for routine quality control of LNPs formulations.

The selectivity of the proposed multiplexed electrochemical platform was evaluated through both cross-talking experiments between the two target miRNAs and interference studies using noncomplementary miRNA sequences.

To assess potential interference between miR-4676 and miR-6503, different mixtures of the two targets were analyzed while maintaining a constant total miRNA concentration. The following miR-4676:miR-6503 ratios were tested: 0:0, 0:50, 50:50, 20:80, and 80:20 nM. As shown in Figure 4A, each working electrode responded selectively to its complementary.

**Figure 4:**
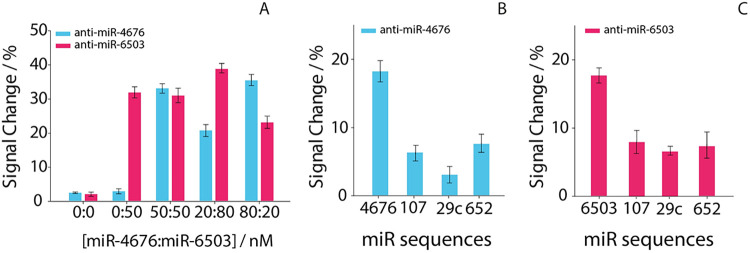
(A) Cross-talking study of the sequence-specific DNA probes using different mixtures of miR-4676 (blue bars) and miR-6503 (pink bars). Signal variations observed in mixed-miRNA samples reflect comparable target concentrations rather than probe cross-reactivity. (B) Selectivity study of the miR-4676 probe in the presence of noncomplementary miRNA sequences (20 nM). (C) Selectivity study of the miR-6503 probe in the presence of noncomplementary miRNA sequences (20 nM). All experiments were performed in triplicate.

Selectivity was further assessed by testing noncomplementary miRNA sequences (miR-107, miR-29c, and miR-652) at a concentration of 20 nM. As reported in Figures 4B,4C, all nontarget miRNAs produced signal changes below 10% for both probes, confirming the high sequence specificity of the sensing interface.

Calibration experiments in undiluted human serum were performed to evaluate the robustness of the biosensor in additional complex biological matrices. The miRNA concentration range was selected to match the analytical operating range of the sensor and to enable comparison with LNP-based calibrations, rather than to reflect endogenous circulating miRNA levels. While the overall sigmoidal trends observed in serum and LNP-based matrices are comparable, moderate differences in signal amplitude and slope are evident. These differences can be attributed to the distinct matrix compositions: LNP-based samples contain residual lipid components and surfactant (Triton X-100) following nanoparticle lysis, whereas serum is a protein-rich environment, which may differently affect surface accessibility and hybridization kinetics. Despite these matrix-dependent effects, the dynamic range and analytical sensitivity of the biosensor are preserved. Under these conditions, limits of detection of 2.9 nM for miR-4676 and 3.88 nM for miR-6503 were obtained (Figure S5, SI).

Following the analytical validation, the platform was employed for the quantification of total miRNA encapsulated within LNPs formulations containing miR-4676 and miR-6503 in two different ratios (1:1 and 2:1). Prior to electrochemical analysis, LNP samples were diluted (1:500) to ensure operation within the linear dynamic range of the signal-off biosensor. At higher miRNA concentrations (above approximately 1 μM), the platform reaches the signal plateau so the applied dilution allows accurate and reproducible quantification within the most reliable response region of the sensor. The results were compared with those obtained by a standard fluorimetric assay. To perform the quantification, serial dilutions of the initial LNPs formulations were tested. The electrochemical response was then compared with that of spiked empty LNPs. The calibration curves obtained from both individual and mixed miRNA-loaded formulations are shown in Figure 5.

**Figure 5:**
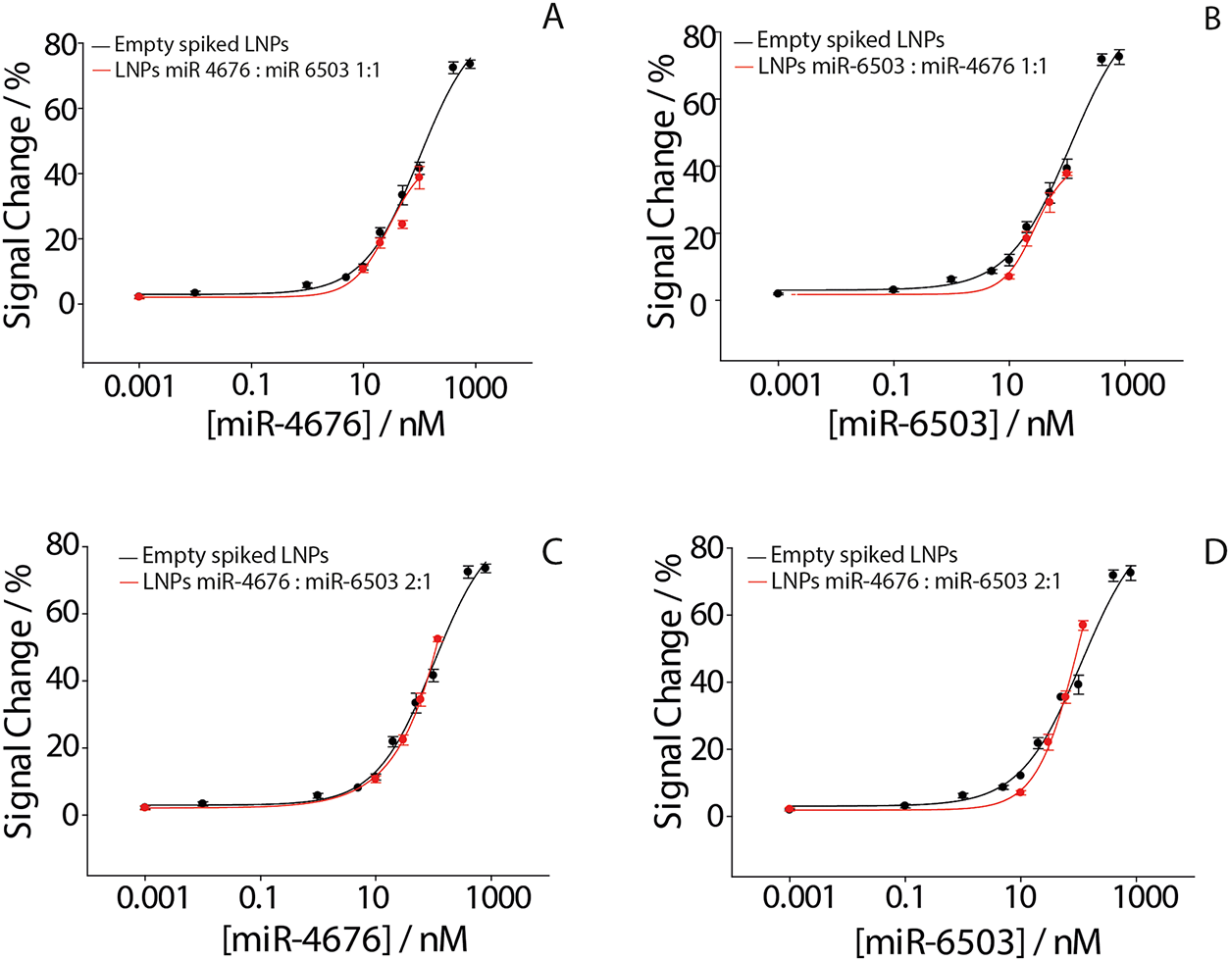
Comparison between the calibration curves obtained for empty spiked LNPs (black) and for miRNA- loaded LNPs with a concentration ratio of 1:1 and 2:1 formulation (red). (A) miR-4676 in LNPs formulation ratio 1:1; (B) miR6503 in LNPs formulation ratio 1:1; (C) miR-4676 in LNPs formulation ratio 2:1; (D) miR-4676 in LNPs formulation ratio 2:1. All the measurements were performed in the presence of Triton X-100 and in triplicate.

For the 1:1 formulation (Figure 5A,B), the total miRNA concentration measured by the standard method was 19 ± 3 μM, while the electrochemical sensor quantified 16.7 ± 0.6 μM, resulting in an accuracy of 88%. Individual quantification yielded 8.6 ± 0.4 μM for miR-4676 and 8.1 ± 0.8 μM for miR-6503.

For the 2:1 formulation (Figure 5C,D), the standard method detected 19 ± 4.8 μM, while the biosensor measured 16 ± 1.4 μM, corresponding to an accuracy of 84%. The biosensor quantified 5.2 ± 0.8 μM of miR-4676 and 10.9 ± 1.9 μM of miR-6503. These results confirm the sensor’s capability to accurately quantify multiple miRNA targets encapsulated in LNPs formulations, supporting its potential application in pharmaceutical quality control. A concise comparison of the main analytical and practical features of the proposed biosensor with other representative methods used for miRNA or LNPs analysis is reported in Table S1 (SI), highlighting the balance between, sensitivity, assay simplicity and suitability for routine quality control.

## Conclusions

In this study, a dual-working electrochemical biosensor was developed, optimized and validated for the multiplex detection of two microRNAs, miR-4676 and miR-6503, encapsulated in LNPs formulations for pharmaceutical applications. To adapt the sensor to graphite-based electrodes and ensure compatibility with surfactant-mediated LNPs disruption, gold electrodeposition was introduced and optimized, yielding improved probe stability and signal retention in the presence of Triton X-100. Calibration curves demonstrated excellent analytical performance of the proposed dual biosensor, with limits of detection of 1.5 nM for miR-4676 and 1.9 nM for miR-6503. The system also exhibited high specificity and negligible cross-reactivity between targets. Unlike standard fluorimetric methods, such as the RiboGreen assay, which provide only the total miRNA concentration without discriminating between individual sequences, the developed biosensor enabled separate quantification of each miRNA species within the same LNP formulation. In two different miRNA mixtures encapsulated in LNP formulations (1:1 and 2:1 miR-4676:miR-6503), the total concentration measured electrochemically closely matched the RiboGreen values, with overall quantification accuracies up to 88%. Although LNP formulations typically contain miRNAs at micromolar concentrations, the use of a controlled dilution step to operate within the linear dynamic range of the signal-off biosensor is therefore fully consistent with standard pharmaceutical quality-control workflows and does not compromise the relevance or accuracy of the analysis.

These results highlight the strong potential of this amplification-free, miniaturized, and multiplexed electrochemical biosensor as a reliable and cost-effective tool for the selective quantification of miRNAs in complex LNPs formulations. To our knowledge, this work represents the first application of a multiplexed electrochemical biosensing strategy to the quantitative analysis of multiple miRNA targets coencapsulated within lipid nanoparticles, specifically addressing pharmaceutical quality-control needs. The sensor demonstrated high sensitivity, excellent specificity, and strong agreement with standard fluorimetric methods, while offering the added advantages of individual miRNA discrimination, operational simplicity, and compatibility with routine sample handling. These features make it ideally suited for integration into both research and industrial workflows, supporting rigorous quality control and accelerating the development of RNA-based therapeutics. In addition, the use of low-cost, disposable screen-printed electrodes, a compact potentiostat and custom 3D-printed cells makes the platform intrinsically amenable to miniaturization and deployment outside conventional laboratory settings. These characteristics, together with the multiplexed format and amplification-free operation, make the biosensor a promising candidate for at-line or near-line quality control of LNP formulations, including in resource-limited or fast-paced manufacturing environments. Despite its promising performance, some aspects of the present platform could be further improved. For instance, the current configuration requires a pretreatment step for LNPs disruption. Future work will aim to simplify this process and integrate it into a more automated and user-friendly format. Moreover, expanding the multiplexing capability and validating the biosensor with a broader range of biological samples will further enhance its applicability for clinical and pharmaceutical use.

## Supplementary Material



## Notes

Additional experimental details, material and methods (PDF)
