# Supplementary material for: Multiplex Detection and Quantification of miRNAs in Drug Delivery Systems Using a Signal-Off Electrochemical Platform
Source: Anal Chem. 2026 Apr 27;98(18):13211–9. doi: 10.1021/acs.analchem.5c07348 (PMC13177283; doi:10.1021/acs.analchem.5c07348)
Supplement: Supplementary file 1 [file ac5c07348_si_001.pdf]

Supporting Information

Multiplex detection and quantification of miRNAs in drug delivery systems using a Signal-Off electrochemical platform

Wanda Cimmino[a‡], Alessia Angelillo[a‡], Panagiota M. Kalligosfyri[a], Valeria Nele[a], Virginia Campani[b*], Stefania Carbone[c], Concetta Di Natale[c], Giuseppe De Rosa[a], and Stefano Cinti[a,d,e,f*]

a Department of Pharmacy, University of Naples Federico II, 80131 Naples, Italy.
b Department of Life Science, Health and Health Professions, Link Campus University, Rome 00165, Italy
c Department of Chemical, Materials and Industrial Production Engineering, University of Naples Federico II, Piazzale V. Tecchio 80, Naples, 80131, Italy.
d Bioelectronics Task Force at University of Naples Federico II, Via Cinthia 21, Naples 80126, Italy.
e Sbarro Institute for Cancer Research and Molecular Medicine, Center for Biotechnology, College of Science and Technology, Temple University, Philadelphia, PA 19122, USA
f Department of Chemistry, Faculty of Science, Chulalongkorn University, Bangkok, Thailand

*** Corresponding author: Virginia Campani v.campani@unilink.it; Stefano Cinti, stefano.cinti@unina.it**

Contents

Abstract	S1
Chemicals and Materials	S2
Characterization of lipid nanoparticles	S2
Surface Morphology and Elemental Characterization of Gold-Deposited Electrodes (SEM–EDX)	S2
Electrochemical evaluation of gold electrodeposition	S3
Analytical Performance in Serum Samples	S4
Comparison between miRNAs quantification methods	S5
References	S6

Abstract

This Supporting Information includes the complementary data related to the characterization and validation of the developed biosensing platform. First, physicochemical properties of the lipid nanoparticles (hydrodynamic diameter, polydispersity index, zeta potential, and encapsulation efficiency) are reported to confirm their suitability for miRNA loading. The morphological and elemental characterization of bare and gold-modified electrodes, assessed by SEM and EDX analysis, is also presented to demonstrate the effectiveness of the electrodeposition step. Furthermore, calibration curves in undiluted human serum are included to confirm the analytical performance in a clinically relevant matrix. Finally, a comparative analysis between the developed biosensor and commonly used techniques for miRNA quantification is provided, highlighting differences in cost, assay time, and operational complexity.

Chemicals and Materials

All chemicals used in this study were purchased from Sigma-Aldrich (St. Louis, MO, USA), including PBS tablets (140 mM NaCl, 10 mM phosphate buffer, 3 mM KCl), 6-mercapto-1-hexanol (MCH, $C_6H_{14}OS$), tris(2-carboxyethyl)phosphine (TCEP, $C_9H_{15}O_6P$), Triton X-100, cloroauric acid ($HAuCl_4$) and potassium ferricyanide $K_3Fe(CN)_6$.

The single-stranded DNA (ssDNA) capture probes targeting miR-6503 and miR-467**6**, both modified with a 5'-thiol-C6 and a MB tag at the 3' end (5'-thiol-C6–MB–3'), were obtained from Metabion GmbH (Steinkirchen, Germany), along with their corresponding synthetic target sequences. MicroRNA 4676 (5'rCrArCrUrGrUrUrUrCrArCrCrArCrUrGrGrCrUrCrUrU-3') and microRNA 6503 (5'-rArGrGrUrCrUrGrCrArUrUrCrArArArUrCrCrCrCrArGrA-3') were synthesized by Tema Ricerca S.r.l. (Bologna, Italy). SM-102 (8-[(2-hydroxyethyl)[6-oxo-6-(undecyloxy)hexyl]amino]-octanoic acid, 1-octylnonyl ester) was purchased by Cayman Chemical (Ann Arbor,USA). DMG-PEG 2000 (1,2-dimyristoyl-rac-glycero-3-methoxypolyethylene glycol-2000) was provided by Avanti Polar Lipids (Alabaster, USA). Disteroylphosphatidylcholine (DSPC) was kindly offered from Lipoid GmbH (Cam, Switzerland). The Quant-iT RiboGreen RNA Assay was supplied from ThermoFisher Scientific (Milan, Italy), while Ethanol and other

solvents were obtained by Exacta Optech (Italy).

Dual screen-printed carbon electrodes (model X1110; WE and AUX: carbon, REF: Ag) were provided by Metrohm DropSens (Oviedo, Spain). Electrochemical measurements were performed using a Metrohm µStat400 potentiostat, with data acquisition and analysis managed through DropView 8400 software. Custom 3D-printed electrochemical cells were fabricated using a Creality Ender-3 V2 Neo 3D printer (Shenzhen Creality 3D Technology, Shenzhen, China).

Characterization of lipid nanoparticles

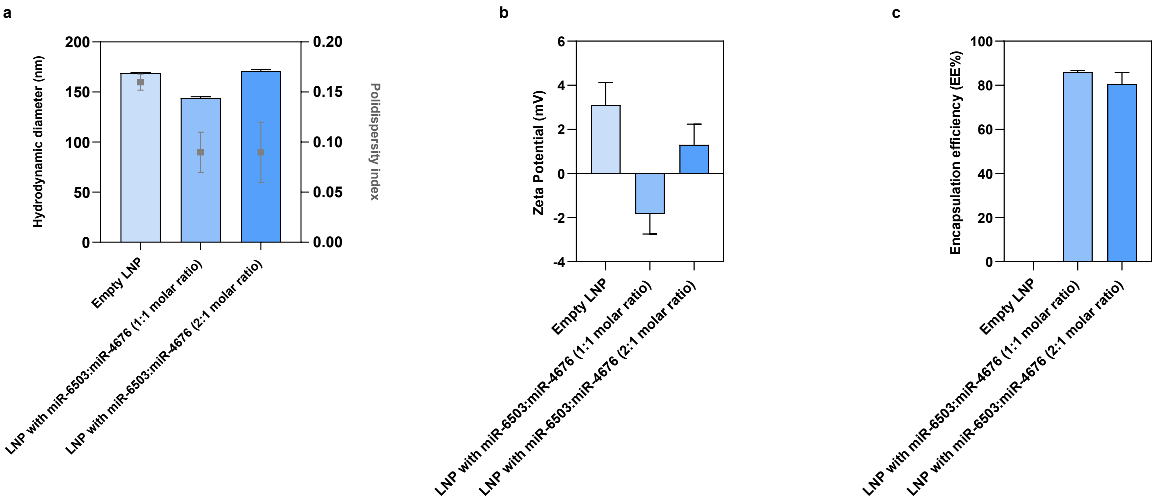

Figure S1: Hydrodynamic diameter and PDI (a), Zeta potential (b) and miRNA Encapsulation efficiency (c) of LNPs Co-encapsulating miR-4676 and miR-6503 (1:1 or 1:2 molar ratio), as well as empty LNPs.

Surface Morphology and Elemental Characterization of Gold-Deposited Electrodes (SEM–EDX)

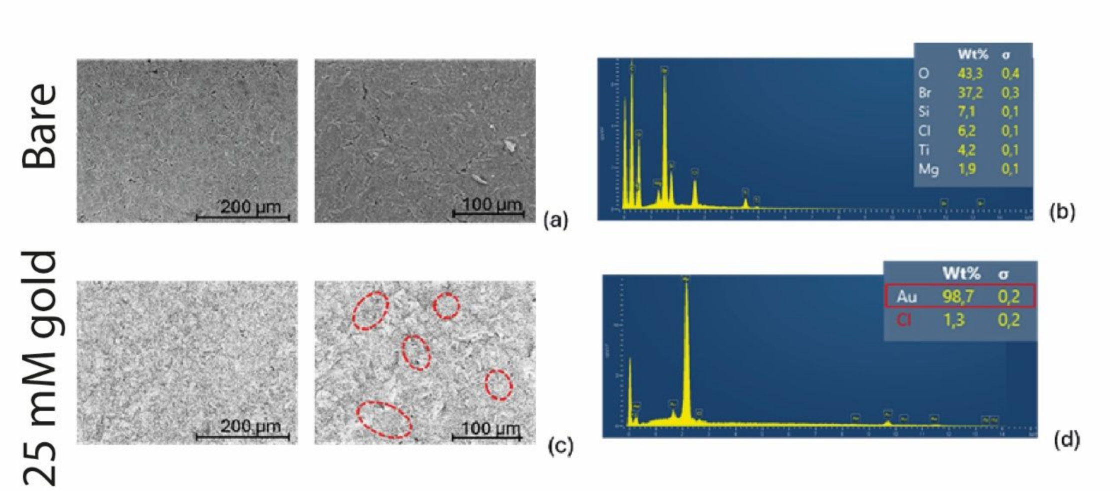

Figure S2: Surface morphology and elemental composition of the bare electrode and the electrode modified with 25 mM gold particles. (a) SEM micrographs of the bare electrode at two magnifications (scale bars: 200 µm and 100 µm) showing a homogeneous surface without visible gold-particle functionalization. (c) SEM micrographs of the gold-modified electrode at the same two magnifications (200 µm and 100 µm) where the dashed red circles highlight discrete gold-particle features on an otherwise uniform background. (b) EDX spectrum of the bare electrode showing no detectable Au signal, confirming absence of gold coverage. (d) EDX spectrum of the gold-modified electrode indicating ~98.7 wt % Au (residual ~1.3 wt % Cl), demonstrating highly efficient and uniform gold electrodeposition.

Figure S2 presents SEM micrographs of the bare electrode (Figure S2 (a), scale bars 200 µm and 100 µm) and the electrode modified with 25-mM gold (Figure S2 (c), same magnifications). For the bare electrode, the surface appears smooth and homogeneous no discrete particles or surface features attributable to gold deposition are visible. This morphological observation is corroborated by the corresponding EDX spectra (Figure S2 (b)), which shows no detectable Au signal, thus confirming the absence of gold functionalization of the bare substrate.

In contrast, the gold-modified electrode (Fig. S2 (c,d)) displays a likewise uniform background morphology, yet discrete features consistent with electrodeposited gold are highlighted by dashed red circles in the 100 µm-scale images. These features indicate that, despite the overall homogeneous appearance, at higher magnification the nanoscopic gold domains can be resolved. The accompanying EDX spectrum (Fig. S2 (d)) reveals a dominant Au peak corresponding to ~98.7 wt% (with residual Cl ~1.3 wt%), thus demonstrating that the gold deposition was highly efficient and the surface is essentially gold-covered. The very high Au weight fraction suggests that ~98% of the analyzed surface area is covered by gold.

Figure S3 further supports this trend, showing EDX spectra of electrodes modified with increasing gold precursor concentration: 2.5 mM (~28.3 wt% Au), 10 mM (~43.2 wt% Au), and 50 mM (~98.9 wt% Au). These results indicate a clear concentration-dependent increase in gold surface coverage.

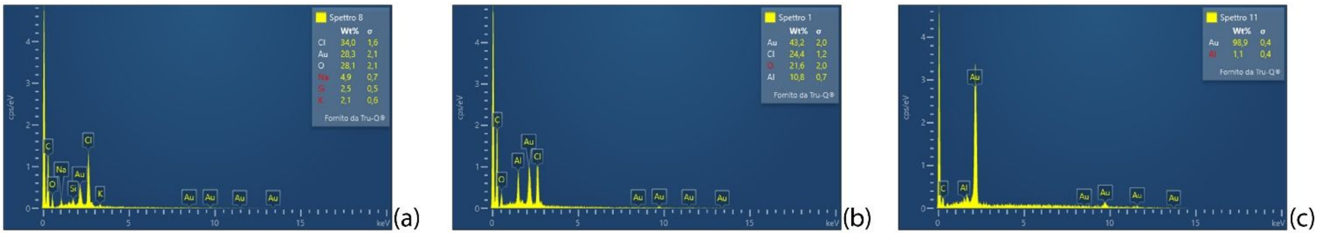

Figure S3: (a) EDX spectrum of the gold-modified electrode (2.5 mM gold) indicating ~28.3 wt % Au, (b) EDX spectrum of the gold-modified electrode (10 mM gold) indicating ~43.2 wt % Au, (c) EDX spectrum of the gold-modified electrode (50 mM gold) indicating ~98.9 wt % Au

Taken together, the SEM and EDX data indicate that the electrodeposited gold layer (25mM) is both morphologically uniform at the micrometer scale and chemically pure. In addition, the combination of morphological homogeneity and elemental purity likely contributes to improved sensor performance via enhanced conductive pathways and stable surface chemistry.

Electrochemical evaluation of gold electrodeposition

The electrochemical behavior of graphite electrodes before and after gold electrodeposition (25 mM) was evaluated by cyclic voltammetry. As shown in Figure S1, gold deposition resulted in a marked increase in the current response, indicating the successful formation of an electroactive gold layer on the electrode surface.

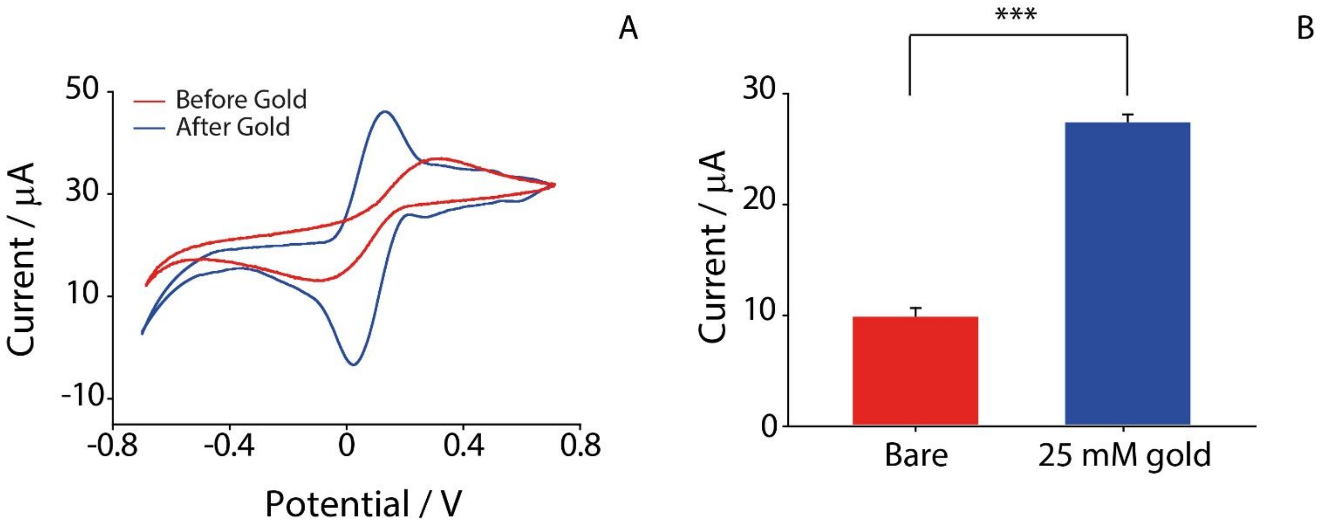

Figure S4: A) Cyclic voltammograms recorded on bare graphite electrodes (red curve) and after gold electrodeposition performed using 25 mM $HAuCl_4$ (blue curve), in the presence of 5 mM of potassium ferricyanide $K_3Fe(CN)_6$. B) Comparison of the anodic peak current measured on bare and gold-deposited graphite electrodes (25 mM $HAuCl_4$). Data shown as mean ± SD, where $n = 3$ statistical significance: *** $p < 0.001$. The electrochemical measurments were performed with the following parameters: E begin: 0.7 V, E vertex1: 0.7 V; E vertex 2: -0.7 V, E step 0.001 V, Scan rate: 0.05 V/s, number of scan: 2.

Analytical Performance in Serum Samples

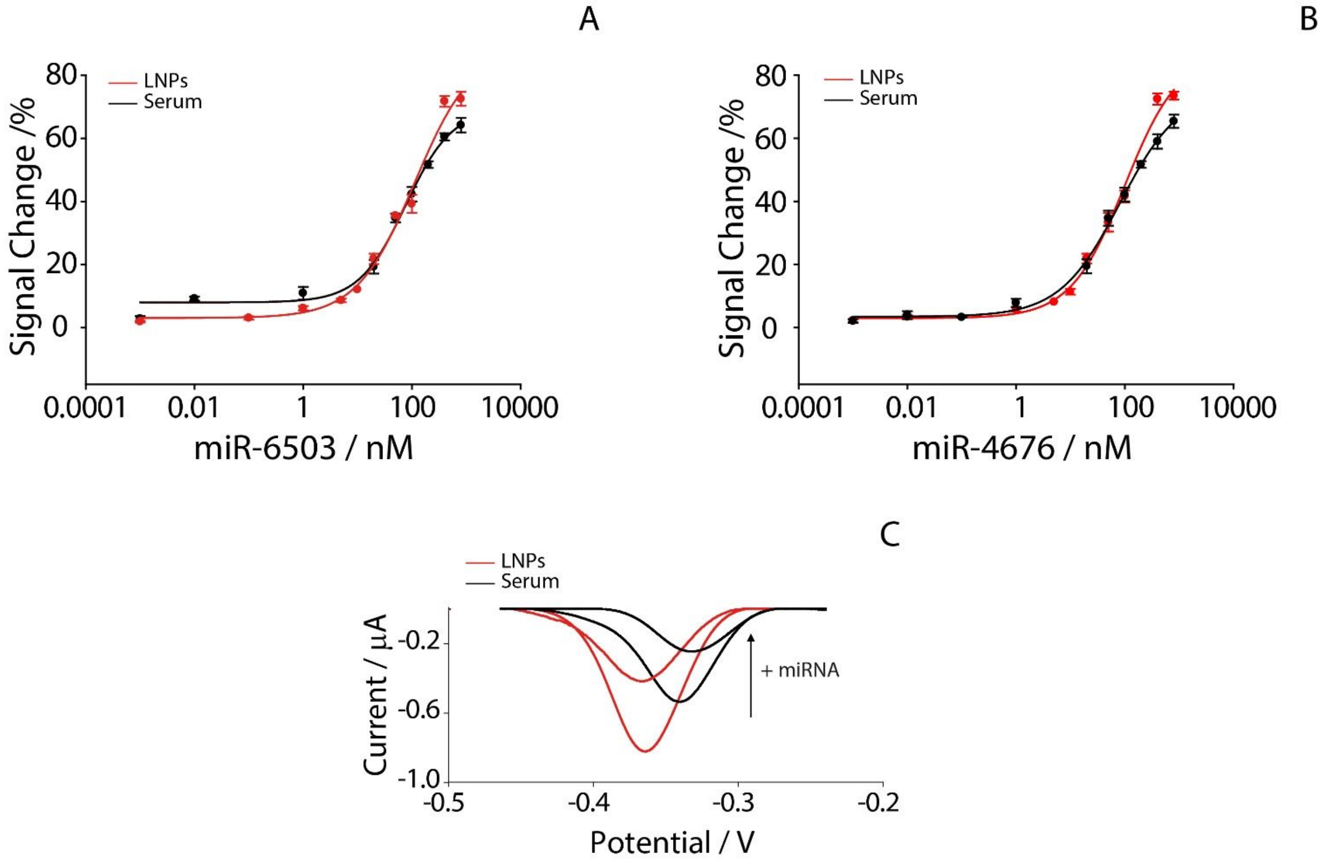

Figure S5: Analytical performance of the platform in LNPs and serum matrices. (A) Calibration curve for miR-6503 obtained using the signal-off electrochemical biosensor in spiked blank LNP (red symbols) and human serum (black symbols). (B) Calibration curve for miR-4676 recorded under the same experimental conditions in spiked blank LNP (red symbols) and serum (black symbols). Signal change (%) is reported as a function of the target miRNA concentration on a logarithmic scale. (C) Representative Square Wave voltammograms recorded before and after target hybridization in LNP (red curveds) and serum (black curves), showing the progressive decrease of the electrochemical signal upon increasing miRNA concentration.

Comparison between the miRNAs quantification methods

Table S1: Comparison between the two methods ad RT qPCR used for the quantification of miRNAs.

Parameter	Electrochemical Biosensor	Quant-iT RiboGreen	RT qPCR
**Unit Cost**	0.20 - 3 € per electrode[1]	5-10€ per test[2]	˜10 € per test

Parameter	Electrochemical Biosensor	Quant-iT RiboGreen	RT qPCR
**Assay Time**	~ 30 min	~ 45 min	~ 2 h
**LOD level**	Low nM	Low pM	~ aM[3]
**Green Score**[4]	80%	64%	45%

References

(1) Kalligosfyri, P. M.; Miglione, A.; Cinti, S. Screen-Printing and 3D-Printing Technologies in Electrochemical (Bio)Sensors: Opportunities, Advantages and Limitations. *ECS Sens. Plus* **2025**, *4* (1), 010601. https://doi.org/10.1149/2754-2726/ada395.

(2) *Quant-it™ RiboGreen Reagent and RNA Assay Kit*. https://www.thermofisher.com/order/catalog/product/R11490 (accessed 2025-04-22).

(3) Androvic, P.; Valihrach, L.; Elling, J.; Sjoback, R.; Kubista, M. Two-Tailed RT-qPCR: A Novel Method for Highly Accurate miRNA Quantification. *Nucleic Acids Res* **2017**, *45* (15), e144. https://doi.org/10.1093/nar/gkx588.

(4) Pena-Pereira, F.; Wojnowski, W.; Tobiszewski, M. AGREE—Analytical GREEnness Metric Approach and Software. *Anal. Chem.* **2020**, *92* (14), 10076–10082. https://doi.org/10.1021/acs.analchem.0c01887.
